# Chemical Characterization of a Collagen-Derived Protein Hydrolysate and Biostimulant Activity Assessment of Its Peptidic Components

**DOI:** 10.1021/acs.jafc.2c04379

**Published:** 2022-08-30

**Authors:** Stefano Ambrosini, Bhakti Prinsi, Anita Zamboni, Luca Espen, Serena Zanzoni, Chiara Santi, Zeno Varanini, Tiziana Pandolfini

**Affiliations:** † Department of Biotechnology, University of Verona, Verona 37134, Italy; ‡ Department of Agricultural and Environmental Sciences - Production, Landscape, Agroenergy, Università degli Studi di Milano, Milan 20133, Italy; § Centro Piattaforme Tecnologiche, University of Verona, Verona 37134, Italy

**Keywords:** biostimulants, bioactive peptides, root growth, mass spectrometry, sustainable agriculture

## Abstract

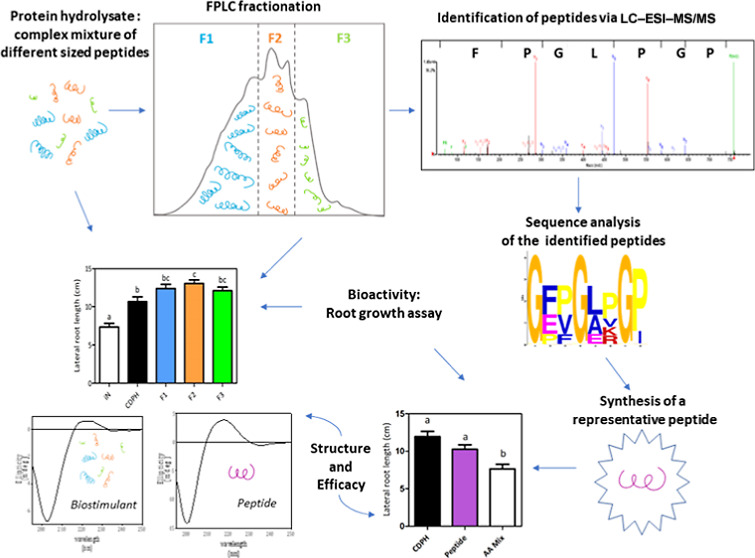

Protein hydrolysates (PHs) are plant biostimulants consisting of oligopeptides and free amino acids exploited in agriculture to increase crop productivity. This work aimed to fractionate a commercial collagen-derived protein hydrolysate (CDPH) according to the molecular mass of the peptides and evaluate the bioactivity of different components. First, the CDPH was dialyzed and/or filtrated and analyzed on maize, showing that smaller compounds were particularly active in stimulating lateral root growth. The CDPH was then fractionated through fast protein liquid chromatography and tested on *in vitro* grown tomatoes proving that all the fractions were bioactive. Furthermore, these fractions were characterized by liquid chromatography–electrospray ionization–tandem mass spectrometry revealing a consensus sequence shared among the identified peptides. Based on this sequence, a synthetic peptide was produced. We assessed its structural similarity with the CDPH, the collagen, and polyproline type II helix by comparing the respective circular dichroism spectra and for the first time, we proved that a signature peptide was as bioactive as the whole CDPH.

## Introduction

Agricultural production will face in the future major challenges all over the world to sustain population growth without compromising environmental health and safety. To reduce the massive use of fertilizers and pesticides, it is necessary to develop novel agriculture practices and more efficient environmentally friendly compounds. In this regard, substances that act at low concentrations to induce plant metabolic responses could be valuable tools to improve crop fitness and/or resilience. Biostimulants are products used at low dosage to stimulate nutrient uptake and assimilation, to improve stress tolerance or quality traits regardless of their nutrient content.^1−4^ Protein hydrolysates (PHs), humic substances, chitin and chitosan derivatives, plant growth promoting bacteria, seaweed extracts,^5,6^ and many other products, as unrelated as they might seem, are all classified as biostimulants when they meet the abovementioned criteria.

Although these substances are extremely heterogeneous in their biochemical composition and are produced with processes very different from one another, they often exert similar beneficial effects on plant growth and stress resistance, most likely related to different mechanism(s) of action, which are indeed specific for each class of biostimulants.^7^

The physiological processes improved by the application of biostimulants are often related with the stimulation of enzymes involved in N uptake and assimilation, the increase in micronutrient accumulation, a rewired hormonal activity, that is, the induction of phytohormone synthesis or a modified hormonal signaling, and protection from oxidative stress.^3,8−13^ However, because these products are particularly complex matrices, knowledge of their mode of action at the biochemical and molecular levels is still largely elusive and scarce information is available on the nature of the active molecules responsible for their biostimulant activity.^14^ The application of a reductionist approach seems convenient to identify the active components of a biostimulant and to study their mechanisms of action. Only in a very few studies, fractionation and chemical analysis have been performed to characterize the bioactivity of some components of a biostimulant.^15−17^ These types of investigations are crucial to gather more insights to ameliorate the industrial process, to optimize the product formulations, or to suggest a different mode of application on crops.^14,18^ Furthermore, unraveling the mechanism(s) of action might provide some hints on where to look for new matrices to exploit, and which contain the identified or similar bioactive compounds. Lastly, in an ever-changing regulatory framework, these details can be crucial to better place and present the product in the market.

For some biostimulants, such as those consisting of plant and seaweed extracts or plant growth promoting bacteria inocula or consortia, pinpointing the bioactive compound(s) of the matrix is a challenging task, due to the incredible variety of biomolecules that they contain, whereas PHs derived from tissues that contain a predominant type of protein (*e.g.*, collagen- or keratin-rich tissues) could be exploited as a simpler study model.^18^

PHs are mixtures of peptides and free amino acids (AAs) obtained from plant or animal tissues through industrial processes using proteases or chemical treatments. Both the length of the peptides and the percentage of free AAs can vary depending on the hydrolytic process applied. Free AAs and small peptides are by far the most abundant and therefore the primary bioactive components of PH-based formulations, as other molecules and mineral elements may be present but only in trace amounts.^18^

Plant roots can absorb N in these organic forms from the external medium and distribute them to different organs through the activity of AA and peptide transporter proteins.^19^ AAs and peptides can affect plant performance in several ways, as they can be used in plant cells as a source of N in biosynthetic processes or they can participate in cell-to-cell and systemic signaling that controls growth and development.^20−23^

Collagen-derived PHs (CDPHs), which are produced using animal connective tissues as raw material, account for a large proportion of the commercial animal-derived PHs. CDPHs supplied either by foliar application or soil drenching promote crop growth and development.^8,24^ In hydroponically grown plants, CDPHs stimulate root growth, often increasing the number of root hairs, and cause changes in root architecture, for example, increasing lateral root development rather than primary root growth.^8,24^

Several pieces of evidence suggest that the effects produced by CDPHs on the root system are associated with the activity of the peptides as signal molecules. First, low concentrations of CDPHs that promote root growth cannot be compatible with a “fertilization” effect; and second, a free AA mixture that mimics the composition of CDPH and contains the same total amount of N has has demonstrated a lowerability to enhance root growth than CDPH.^8^ In addition, several transcriptomic and proteomic analyses have indicated that CDPHs can modify the expression of genes involved in nutrient uptake and affect hormone signaling pathways and the response to oxidative stress.^8,9,11^

We have previously characterized the biological effects of a commercial CDPH (produced by the SICIT group) showing that at low concentrations (*i.e.*, total N about 5–15 mg·L^–1^) it causes a remarkable enhancement of maize root growth, increases the uptake of several mineral nutrients, and acts as a protectant against drought, hypoxic stress, and Fe deficiency stress.^3,8^

The aim of this study was to evaluate the plant growth stimulatory activity of the different fractions of the CDPH, subdivided based on their molecular size (AAs and peptides of different lengths), and to assess the nature of the bioactive peptides. We have chemically characterized the CDPH by applying different methods of fractioning and circular dichroism (CD) and we have used mass spectrometry (MS) to identify the peptide species present in the product. The biological activity of the different fractions was evaluated using a root growth assay. Bioinformatic analysis of the identified peptides allowed us to define a highly conserved consensus sequence that represents the hallmark of CDPH peptides. We demonstrated that an artificially synthesized peptide containing this consensus sequence was able to promote root growth in a similar manner as the whole product, showing for the first time that it is possible to find a signature peptide representative of the biostimulant effect of the CDPH.

## Materials and Methods

### Plant Material, Growth Conditions, and Root Growth Analysis

Maize plants were grown as described by Santi *et al.*,(2017).^8^ Briefly, maize seeds (P0423 Hybrid, Pioneer Italia S.p.A.) were soaked in water and germinated in the dark for 72 h. After germination, the seedlings were transferred in a 0.05 mM CaSO_4_ solution for 24 h and then grown in a diluted nutrient solution (100 μM MgSO_4_, 5 μM KCl, 200 μM K_2_SO_4_, 175 μM KH_2_PO_4_, 400 μM CaSO_4_, 25 μM NH_4_H_2_PO_4_, 2.5 μM H_3_BO_3_, 0.2 μM MnSO_4_, 0.2 μM, ZnSO_4_, 0.05 μM CuSO_4_, 0.05 μM NaMoO_4_, 2 μM Fe-EDTA)^25^ under a 16/8 h light/dark regime at 22–26 °C and 125 μE·m^–2^·s^–1^ light intensity. The nutrient solution was supplemented with unfractionated CDPH (SICIT Group) or with CDPH fractions obtained by dialysis and membrane filtration after volume equilibration. After 3 days of treatment, the roots were sampled for root growth analyses. The chemical composition of the CDPH, including the amino acidic profile, has been previously reported.^8^

For the *in vitro* experiments, tomato seeds (Roma VF, Blumen Group S.p.A.) were germinated in 8 g·L^–1^ agar plates. When the root was around 2 cm long, the seedlings were grown vertically in 8 g·L^–1^ agar plates with the appropriate treatment. Plates for both, germination and growth, were kept in a controlled growth chamber at 25 °C with a 16 h light/8 h dark photoperiod, with an average light intensity of 120 μE· m^–2^ s^–1^. Each treatment, that is, inorganic N (iN) (NH_4_H_2_PO_4_), the whole CDPH, the fractionized CDPH (either F1, F2 or F3), the synthetic peptide (Pep) QGLLGApGFLGLpG (p, hydroxyproline) (GenScript), or an AA mix mimicking the peptide composition was normalized on the total content of N (*N*_tot_ = 1.435 mg·L^–1^). After 6 days, the seedlings were collected for root growth analysis.

Maize and tomato root growth analysis was performed by using WinRHIZO scanner and automated software.^26^ The total N content of the CDPH and the fractions was estimated using the commercial LCK 338 LATON kit (Hach Lange).

### Filtration, Dialysis, and Fractionation

The CDPH (1:10 diluted) was filtrated with Amicon Ultra-15 centrifugal filter devices (Millipore) with a cutoff of 3 kDa and inserted in 50 mL centrifuge tubes following the manufacturer’s instructions. The dialysis of the whole CDPH or the filtrated CDPH was performed in distilled water in the 20 mL Pur-A-Lyzer tubes (molecular mass cutoff 1 kDa, Sigma Aldrich) as described by the manufacturer.

Fast protein liquid chromatography (FPLC) fractionation of the CDPH was carried out on an FPLC GE Healthcare AKTA pure (GE Healthcare) system. All FPLC runs were performed at room temperature and the elution profile was monitored observing the absorbance values at 214 nm. Size-exclusion chromatography was carried out on a single Superdex 30 Increase 10/300 GL column (GE Healthcare) eluting 0.5 mL of the 1:100 CDPH in a phosphate-buffered saline (PBS) degassed solution (20 mM Na_2_HPO_4_, 20 mM NaH_2_PO_4_, 150 mM NaCl, pH = 7.4) at a 0.5 mL· min^–1^ flow. Similarly, a run was performed to obtain a reference spectrum co-eluting six different molecular standards, that is, 0.05 mg·mL^–1^ bovine serum albumin (*M*_r_ 66 463), 0.2 mg·mL^–1^ cytochrome C (*M*_r_ 12 400), 0.2 mg·mL^–1^ aprotinin (*M*_r_ 6500), 0.07 mg·mL^–1^ vitamin B12 (*M*_r_ 1355), 0.2 mg·mL^–1^ tryglycine (*M*_r_ 189), and 14 mg·mL^–1^ glycine (*M*_r_ 75). Prior to each sample injection into the system, one column volume of eluent buffer was run to ensure equilibration of the column.

### Peptide Sequencing by LC–ESI–MS/MS

The liquid chromatography–electrospray ionization–tandem mass spectrometry (LC–ESI–MS/MS) analyses were conducted both on unfractionated CDPH and on fractions obtained from FPLC (F1, F2, and F3). Before analysis, the biostimulant solution was diluted 1:100 in 0.1% (v/v) formic acid (FA). For each sample, an aliquot (150 μL) was cleaned up by means of the Pierce C18 spin columns (Thermo Scientific), according to the manufacturer’s instructions. The samples were dried in a vacuum evaporator and adequately suspended in 0.1% (v/v) FA. An aliquot of sample (about 1 μg of organic N) was analyzed by an Agilent 6520 Q-TOF mass spectrometer equipped with an HPLC chip cube source driven by a 1200 series nano/capillary LC system (Agilent Technologies). The nLC separation was done using a 75 μm × 150 mm column (Zorbax SB, C18, 300 Å), applying a 100-min acetonitrile gradient [from 5% to 50% (v/v)] in 0.1% (v/v) FA at 0.4 μL·min^–1^. The mass spectrometer ran in positive ion mode acquiring 4 MS spectra s^–1^ from 300 to 3000 *m*/*z* (mass to charge). The auto-MS/MS mode was applied in a range of 50 to 3000 *m*/*z* with a maximum of three precursors per cycle and an active exclusion of two spectra for 0.1 min. Peptide identification was performed by protein database searching with Spectrum Mill MS Proteomics Workbench (Rev B.04.00.127, Agilent Technologies). Search parameters were precursor mass tolerance ±20 ppm and product mass tolerance ±50 ppm, with no enzyme, and proline and lysine hydroxylation set as variable modifications. Hydroxyproline was reported by the symbol “p”. The search was done against the database of *Bos taurus* reviewed protein sequences downloaded from UniProt (https://www.uniprot.org/) (6003 *entries*, October 2018), concatenated with the respective reverse one. The threshold used for peptide identification was FDR <0.01 or spectrum mill score ≥9, scored peak intensity % ≥ 70%, difference between forward and reverse scores ≥2, with a mass tolerance of ±10 ppm. Each sample was independently analyzed twice, and only peptides identified in both analyses were accepted.

### Bioinformatic Analysis

AA sequences were compared with sequences in the GenBank database using the BLAST program. Alignments of the peptides were performed with MultAlin (http://multalin.toulouse.inra.fr/multalin/)^27^ and the *consensus* sequence logos were produced by using the tool STREME (https://meme-suite.org/meme/tools/streme) belonging to the MEME suite.^28^ We set the consensus scores as “low” when identity percentage ranged between 30–70% while “high” when it was above 70%. The control sequences for STREME analysis were randomly generated shuffling the input sequences.

### CD Spectral Measurement

CD spectra were recorded with a JASCO J-1500 spectropolarimeter (Japan Spectroscopic Co., Tokyo, Japan), using a quartz cell of 1 mm path length at 25 °C. CD spectra were scanned in the far-ultraviolet range from 195 to 250 nm. Values were measured at an interval of 1 nm, and the spectra obtained were the average of 3 to 5 reads. All the samples were diluted in 1/2 PBS degassed solution (10 mM Na_2_HPO_4_, 10 mM NaH_2_PO_4_, 75 mM NaCl, pH = 7.4), which was also used as the blank reference. Final spectra resulted from the subtraction of their respective blanks and subsequent smoothing. The CD data were expressed in terms of mdeg because it was not possible to operate a normalization based on molarity for the FPLC eluted fractions and the CDPH.

## Results

### Separation of the Different Components of the Protein Hydrolysate

**Figure 1:**
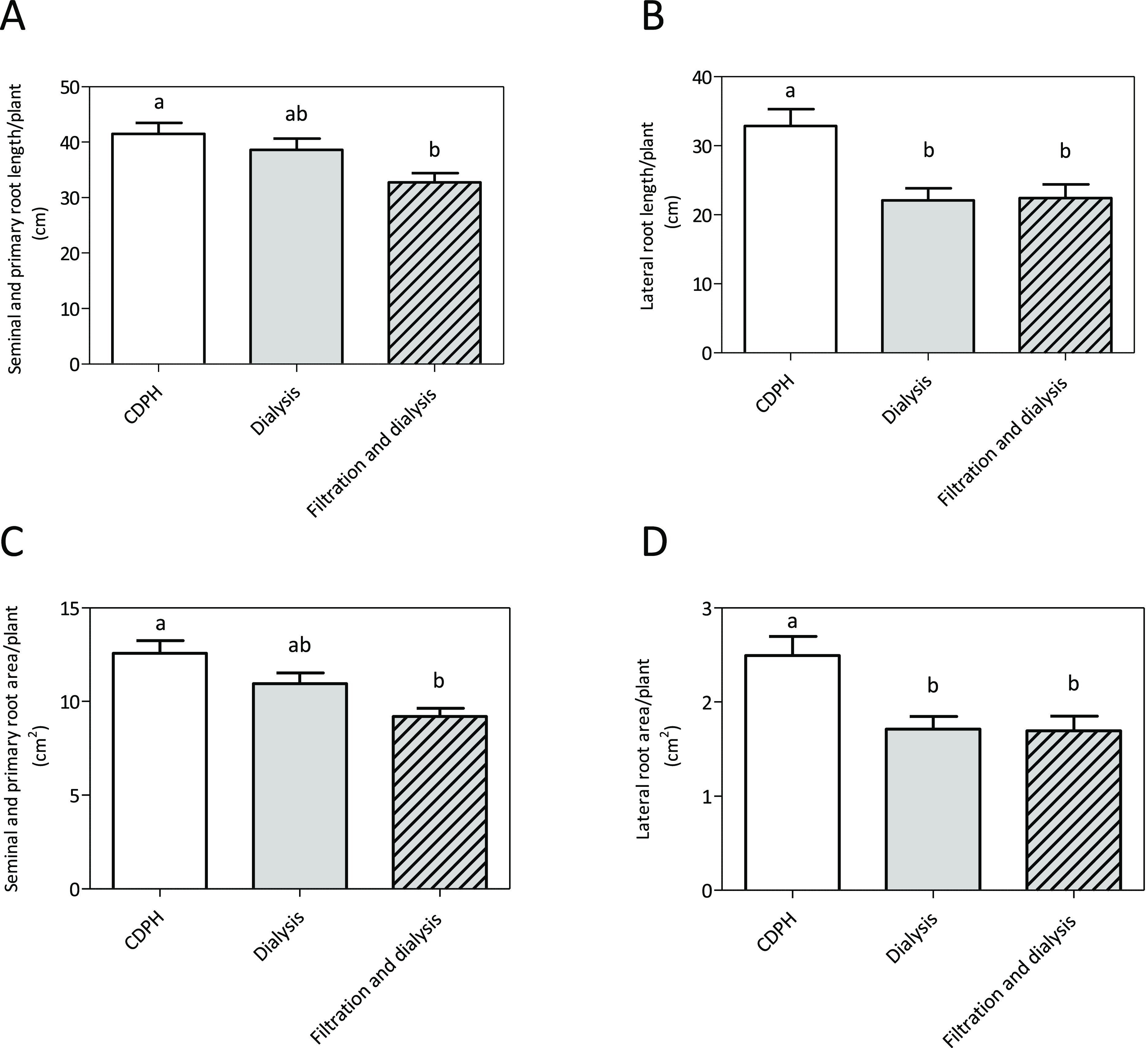
Effects of the CDPH after dialysis and filtration on root growth of maize plants in hydroponic solution. Total seminal and primary root length (A), lateral root length (B), total seminal and primary root area (C), and lateral root area (D) of maize seedlings grown in a nutrient solution supplied with unfractionated CDPH (1:10 000 final dilution), dialysed CDPH and filtrated and dialysed CDPH fractions. The seedlings were grown hydroponically for 7 days. Root length and root area were measured with WinRHIZO software. Mean values per plant are reported. Bars represent the standard error of the mean (*n* ≥ 15 replicates, one-way ANOVA with Tukey’s post hoc test, *p* < 0.05, significant differences are indicated by different letters). Here, we show one experiment representative of two replicates.

The CDPH characterized in this study is produced through the chemical hydrolysis of shavings and trimmings residues of the tanning industry. The CDPH contains 10% of free AAs and a mixture of peptides of different sizes. To determine the contribution of the different components to the bioactivity of the product, we first dialyzed the CDPH to eliminate free AAs and very short peptides (approx. up to 10 AAs). Second, the CDPH was membrane filtered to remove peptides bigger than 3000 Da and then dialyzed to remove free AAs and very short peptides in order to maintain the fraction of peptide between 1000 and 3000 Da. The biological activity of these two preparations (dialysis and filtration and dialysis) was tested in hydroponics. Their effects were evaluated on root morphology of maize seedlings. Data showed that both the separation procedures did not enhance the biostimulant effects exhibited by the whole product. The removal of free AAs and very short peptides did not cause a reduction in seminal and primary root length and area (dialysis, Figure 1A,C) maintaining the biostimulant capacity of the CDPH, even though the subtraction of the smaller components caused a considerable reduction in total N content (Table S1). The removal of the bigger peptides coupled with dialysis (filtration and dialysis) significantly reduced both seminal and primary root length and area. On the other hand, when the product was dialyzed (with or without filtration), a reduction on lateral root growth was observed (Figure 1B,D) suggesting that the main contribution to this effect might be ascribed to these smaller molecules (less than 1000 Da).

To better characterize the type of peptides (in terms of sequence and length) present in the CDPH, we then applied a chromatographic separation based on size exclusion.

**Figure 2:**
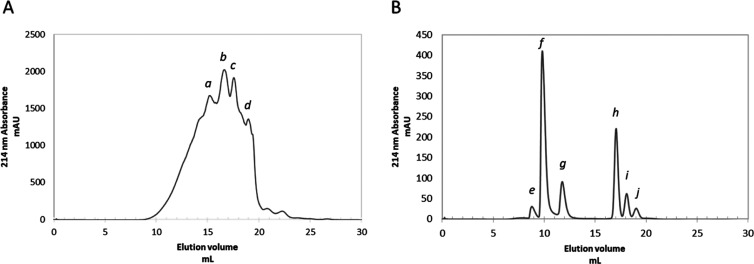
FPLC elution profile of the CDPH. FPLC elution profile of the CDPH in PBS (A) and of the molecular standards (B). The discernible peaks of the CDPH are identified with the letters *a*, *b*, *c*, and *d*, and eluted at 15.0, 16.8, 17.6, and 19.0 mL, respectively. The molecular standards eluted at 8.8 (BSA), 9.8 (cytochrome C), 11.8 (aprotinin), 17.1 (vitamin B12), 18.1 (triglycine), and 19.0 (glycine) mL. All samples were eluted in PBS at 0.5 mL·min^–1^ flow rate. Absorbance values were detected at 214 nm. BSA (peak *e, M*_r_ 66 463 Da), cytochrome C (peak *f, M*_r_ 12 400 Da), aprotinin (peak *g, M*_r_ 6500 Da), vitamin B12 (peak *h, M*_r_ 1355 Da), triglycine (peak *I, M*_r_ 189 Da), and glycine (peak *j, M*_r_ 75 Da).

The size-exclusion chromatographic profile of the product (diluted 1:100) eluted in PBS (PBS: Na_2_HPO_4_ 20 mM, NaH_2_PO_4_ 20 mM, NaCl 150 mM), obtained recording the absorbance at 214 nm, is reported in Figure 2A. Three major peaks were observed corresponding to elution volumes of approximately 15.0, 16.8, and 17.6 mL (respectively named *a*, *b*, and *c*). Another peak (*d*) was clearly distinguishable at 19 mL of eluate. The fractioning was performed several times using different dilutions and flow rates, as well as different batches of the product, with negligible changes in the elution profile (data not shown). Comparing the elution profile of the CDPH with those of several standard proteins, we observed that peptides greater than 5000 Da and di- or tripeptide and free AAs (MW lower than 200 Da) represent minor fractions of the mixture, whereas peptides greater than 6000 Da are virtually/almost absent (Figure 2A,B). The two central peaks are most likely comprised in a range of molecular mass of 200–2000 Da.

**Figure 3:**
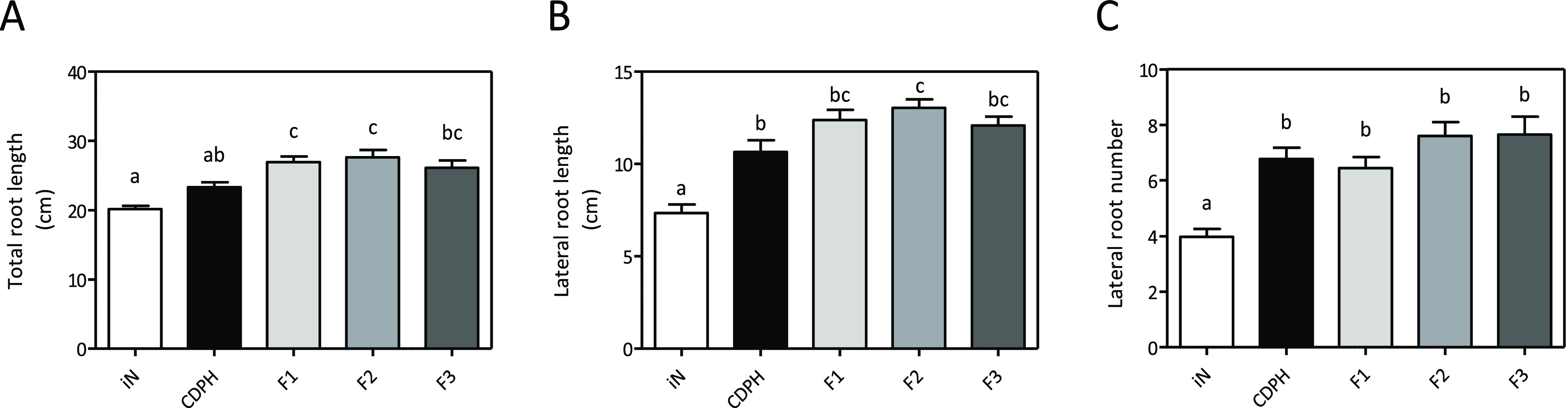
Effects of the CDPH fractions on root growth of tomato seedlings *in vitro* agar media. Total root length (A), lateral root length (B), and lateral root number (C) of tomato seedlings treated with CDPH and seedlings treated with the CDPH fractions (F1, F2, and F3) containing an equal amount of N (1.4 mg·L^–1^). iN, control seedlings treated with NH_4_H_2_PO_4_. The seedlings were grown for 6 days. Root length was measured with WinRHIZO software. Mean values per plant are reported. Bars represent the standard error of the mean (*n* ≥ 29 replicates, one-way ANOVA with Tukey’s post hoc test, *p* < 0.05, significant differences are indicated by different letters). Here, we show the merged data from two independent replicates.

We collected three fractions to test their biostimulant activity and for tandem mass spectrometry (MS/MS) characterization. The first fraction (F1) comprises the peak *a* and heavier peptides, the second one (F2) comprises peaks *b* and *c*, and the third one (F3) peak *d* and free AAs. Moreover, before MS/MS analysis, we verified the preservation of biostimulant activity in the three collected fractions. Because the fractions obtained by gel filtration were very dilute, we developed a system to test the effects of low amount of CDPH on seedlings growing on a solid medium. We observed that by growing *in vitro* young seedlings of different species (*e.g.,* lettuce, basil, and tomato), it was possible to detect a stimulatory effect on root growth after the application of CDPH (corresponding to 14.3 mg·L^–1^ of total N) added to the medium (Supporting Information, Figure 1). We also conducted some experiments at lower concentrations of inorganic N (1.43 and 7.2 mg·L^–1^ NH_4_H_2_PO_4_) in agar alone or in agar containing 1:3 Murashige and Skoog solution. Tomato seedlings treated with the lowest dose of N showed no significant differences in lateral root growth (Supporting Information, Figure 2) and aerial part development (data not shown) when grown with or without MS. On the other hand, a 5-fold higher NH_4_H_2_PO_4_ concentration in the solution containing agar solely proved to be inhibitory for lateral root growth (Supporting Information, Figure 2). We therefore carried out a root growth test on tomato seedlings treated with the three FPLC fractions, the unfractioned CDPH, and inorganic N (NH_4_H_2_PO_4_), equalizing all treatments for the same amount of total N (1.43 mg·L^–1^) (Figure 3).

We observed a stimulatory effect on root growth in terms of total root length, number, and length of lateral roots in the plants treated with each fraction as well as with the unfractionated product in comparison with plants supplied with inorganic N (Figure 3A–C). However, fraction 2 contains peptides that show to be particularly effective in promoting lateral root length (Figure 3B).

### Identification of Peptides Present in the Unfractionated and Fractionated CDPH

**Figure 4:**
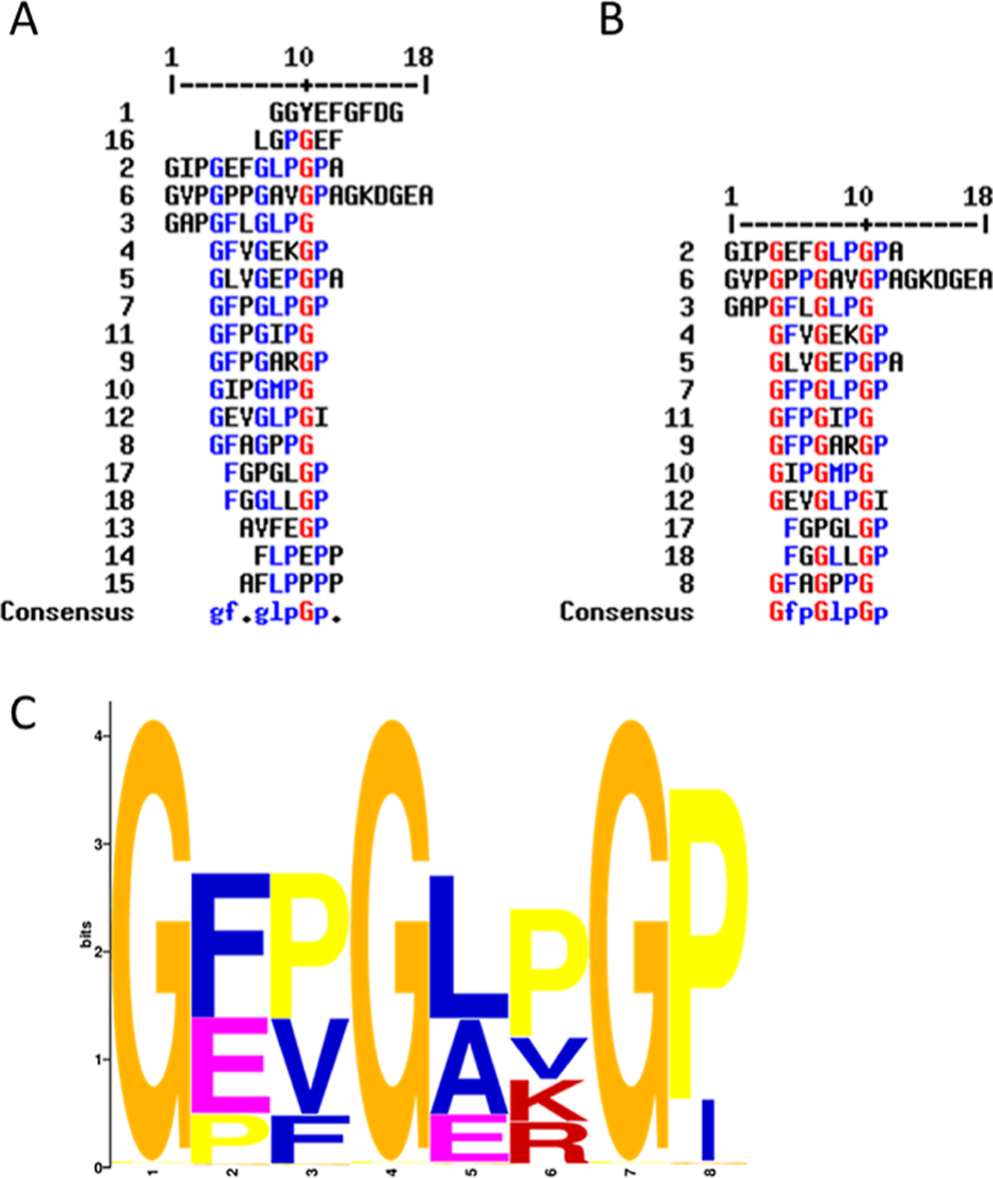
Multiple sequence alignment and sequence logo of the peptides identified *via* LC–ESI–MS/MS. Multiple sequence alignment was carried out by using MultAlin (http://multalin.toulouse.inra.fr/multalin/) online software (A,B).^27^ Sequence logo was produced by using STREME.^28^ The first alignment (A) comprehends all the 18 sequences identified, whereas the second analysis (B) was manually curated in order to eliminate those that differed the most from the others. In the alignments, blue residues indicate low consensus sequence (score comprised between 30 and 70%) whereas red residues indicate high consensus sequence (score above 70%). The same logo (C) was obtained from the analysis of the sequences reported in A (score 1.1e-0031) and B (score 7.0e-004).

MS/MS analysis was carried out on both the diluted unfractionated CDPH and on FPLC fractions. We overall detected 32 peptides of *B. taurus* proteins (Supporting Information, Table 1): 6 were identified in the unfractionated CDPH and 6, 17, and 3 in the FPLC fraction 1, 2, and 3, respectively. The majority of the peptides are fragments derived from three types of collagen (I, IV, and XVII): 16 from alfa 2(1) chain and 5, 3, and 1 from alfa 1(1), 1(IV), and 1(XVII) chains, respectively (Supporting Information, Table 1, Figure 4). We also detected a few peptides from other bovine proteins (*i.e.*, seminal plasma protein, homeobox protein prophet of Pit-1, acetyl-CoA carboxylase 1, phosphatidylinositol 5-phosphate 4-kinase type-2 gamma, xylosyltransferase 2, and antigen WC1.1) (Supporting Information, Table 2, Figure 3). Considering the complexity of the mixture, the identification of a relative low number of peptides in the unfractionated CDPH could be ascribed to a strong matrix effect, partially reduced in the FPLC fractions. Overall, the peptides identified in the fraction F2 had a molecular mass comprised in the range predicted by the chromatographic analysis. We observed that peptides originated from the hydrolysis of the alpha-2 chain of the type I collagen were the most represented and it was evident from the analysis of their sequence that they derived from defined regions of the protein, suggesting possible preferential sites for hydrolytic cleavage (Supporting Information, Figure 3). We identified 18 reference peptide sequences based on the cleavage position and sequence similarity (Table 1). Each one of the reference sequences group together all the MS/MS-identified fragments that were found to match entirely or partially that sequence.

**Table 1:** *B. taurus* Peptides Identified *via* LC–ESI–MS/MS

reference protein (UniProt)^a^	reference peptide sequence^b^	sample^c^	MS identified sequence^d^	starting AA position^e^
COL2A1 (P02465)	**#1. GGYEFGFDG**	CDPH	GYEFGF	1105
		#2	GYEFGFDG	1105
			GYEFGFD	1105
			YEFGFD	1106
			GFDGDFY	1109
		#3	GYEFGF	1105
			GGYEFGF	1104
			YEFGF	1106
	**#2. GIPGEFGLPGPA**	CDPH	GIpGEFGLpG	572
		#1	GIpGEFGLpGPA	572
	**#3. GAPGFLGLPG**	#1	GApGFLGLpG	866
		#2	GFLGLpG	869
			pGFLGLpG	868
	**#4. GFVGEKGP**	#2	GFVGEKG	839
			FVGEKGP	840
	**#5. GLVGEPGPA**	#1	GLVGEpGPA	341
				
COL1A1 (P02453)	**#6. GVPGPPGAVGPAGKDGEA**	#1	GVpGPpGAVGPA	598
			GVpGpPGAVGPAGKDGEA	598
	**#7. GFPGLPGP**	#2	GFPGLpGP	970
	**#8. GFAGPPG**		GFAGPpG	811
	**#9. GFPGARGP**		GFpGARGP	409
				
COL4A1 (Q7SIB2)	**#10. GIPGMPG**	CDPH	GIPGMPG	1088
	**#11. GFPGIPG**	#2	gFpGIpG	362
			GFPGIp	362
				
COL17A1 (A6QPB3)	**#12. GEVGLPGI**	CDPH	GEVGLpGI	683
				
BSP-30K (P81019)	**#13. AVFEGP**	CDPH	AVFEGp	90
		#2	AVFEGp	90
				
PROP-1 (Q8MJI9)	**#14. FLPEPP**	CDPH	FLPEPP	146
				
ACACA (Q9TTS3)	**#15. AFLPPPP**	#1	AFLppPP	1620
				
PIP4K2C (Q0P5F7)	**#16. LGPGEF**	#2	LGpGEF	342
				
Antigen WC1.1 (P30205)	**#17. FGPGLGP**	#2	FGpGLGp	83
				
XYLT2 (Q5QQ49)	**#18. FGGLLGP**	#2	FGGLLGp	664

a Reference protein (UniProt): the acronym of the protein containing the identified peptide with its accession number in the UniProt database.

b Reference peptide sequence: the peptide sequence chosen as representative (in bold).

c Sample: the kind of sample in which the AA sequence(s) was/were identified.

d MS/MS identified sequence: the peptide sequence identified by MS/MS, “p” indicates hydroxyproline.

e The starting position of the MS/MS identified peptide in the reference protein. COL2A1, collagen alpha 2(I) chain; COL1A1, collagen alpha-1(I) chain; COL4A1, collagen alpha-1(IV) chain; COL17A1, collagen alpha-1(XVII) chain; BSP-30K, seminal plasma protein; PROP-1, homeobox protein; ACACA, acetyl-CoA carboxylase; PIP4K2C, phosphatidylinositol 5-phosphate 4-kinase type-2 gamma; antigen WC1.1, antigen WC1.1; and XYLT2, xylosyltransferase 2. CDPH, unfractionated product. #1, #2, and #3, FLP fractions.

The sequence alignment of the 18 representative peptides pointed out a striking similarity also among peptides derived from different proteins (Figure 4A). This is even more evident when we restricted the analysis to the 13 peptides which showed higher similarity (therefore after removing the entry #1, #13, #14, #15, #16 from the list). It was possible to recognize a very similar *consensus* sequence independently of the number (18 or 13) of the representative peptides included in the analysis which differs only in one residue of proline which had a score too low to be accounted as conserved in the alignment of the whole set of peptides (Figure 4A).

The *consensus* sequence logo (Figure 4C) obtained considering the whole set of representative peptides, that is, G(F/E/P)(P/V/F) G (L/A/E) (P/V/K/R) G (P/I), is characterized by the presence of conserved glycines at precise positions in the motif (GxxGxxG). To test the capacity of a single type of peptide to exert biostimulant effects, we chemically synthesized the peptide QGLLGApGFLGLpGS belonging to the collagen alpha 2(I) chain, containing the reference peptide sequence #3. Peptides derived from this region of the alpha 2(I) chain were identified in fraction 1 and 2 (Table 1).

### Biophysical Characterization of the Synthetic Peptide *via* CD Analysis

**Figure 5:**
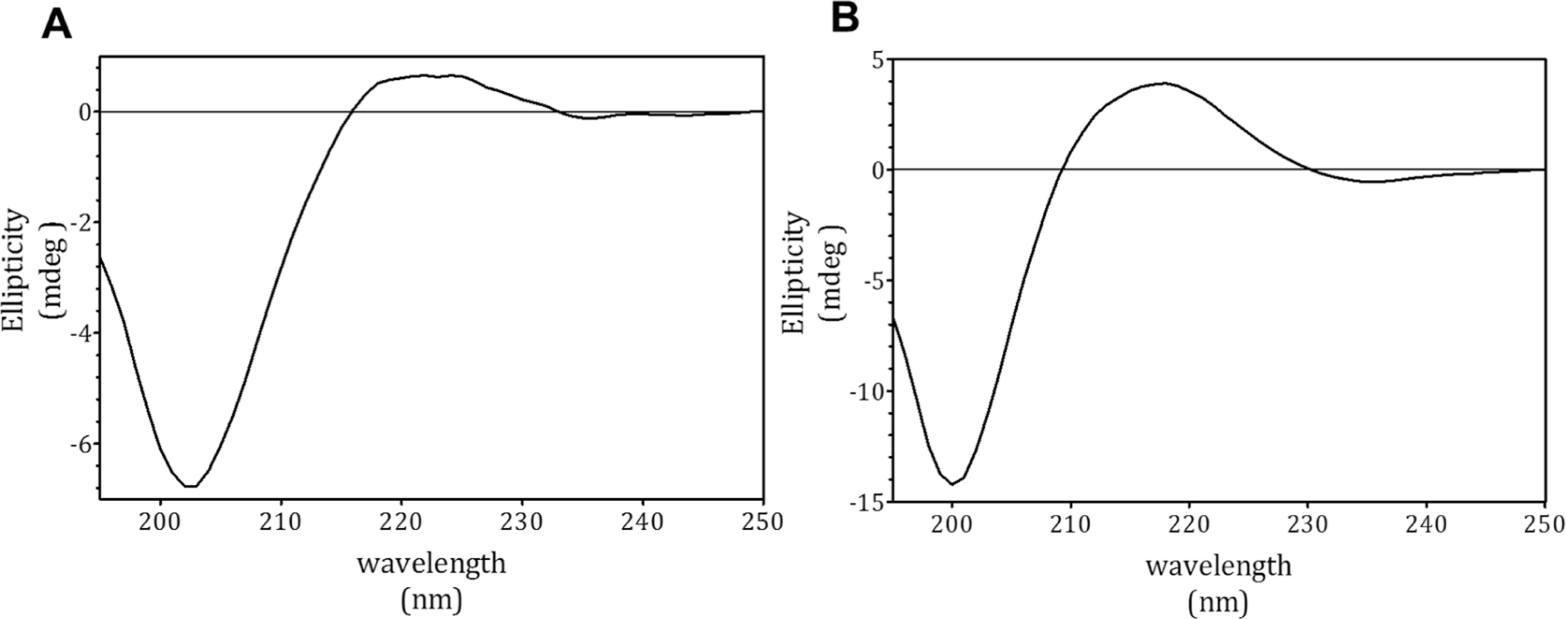
Comparison between the CDPH and the synthetic peptide CD spectra. The figure shows the CD spectra of the CDPH (A) and of the synthetic peptide (B) diluted in 1/2 PBS. Ellipticity was expressed as mdeg.

As previously observed by Ambrosini and co-authors (2021), the CD spectrum of CDPH (Figure 5A) is an intermediate between the typical spectrum of denatured soluble type II collagen and that of polyproline-II (PPII) type spectrum.^29^ The negative minimum of the CDPH was found at 203 nm, between the one at 197 nm of the native collagen and the one at 206 nm of the PPII, whereas the positive maximum was at 222 nm, between the peak at 220 nm of the native collagen and the one at 228 nm of the PPII.^29,30^ Interestingly, the CD spectrum of the short synthetic peptide also strikingly resembles the above cited reference spectra, displaying two peaks: a negative one at 200 nm and a positive one at 218 nm (Figure 5B). Moreover, the CDPH positive peak was quite flattened compared to those of the peptide and of the reference spectra. Among the FPLC-obtained fractions, the spectrum of F1 resembled the CDPH one, showing a minimum at 202 nm and a maximum at 223 nm (Supporting Information, Figure 4), whereas the spectra of F2 and F3 seemed to increasingly lose the characteristic shape, suggesting that longer peptides are responsible for the tridimensional arrangement that is studied with CD analysis. Longer peptides, such as the 15-residues peptide we chose to focus on, are most likely to be found in F1 because the CD spectra of F1 is the most similar to spectra of CDPH (Supporting Information, Figure 4).

### Biological Activity of the Synthetic Peptide

**Figure 6:**
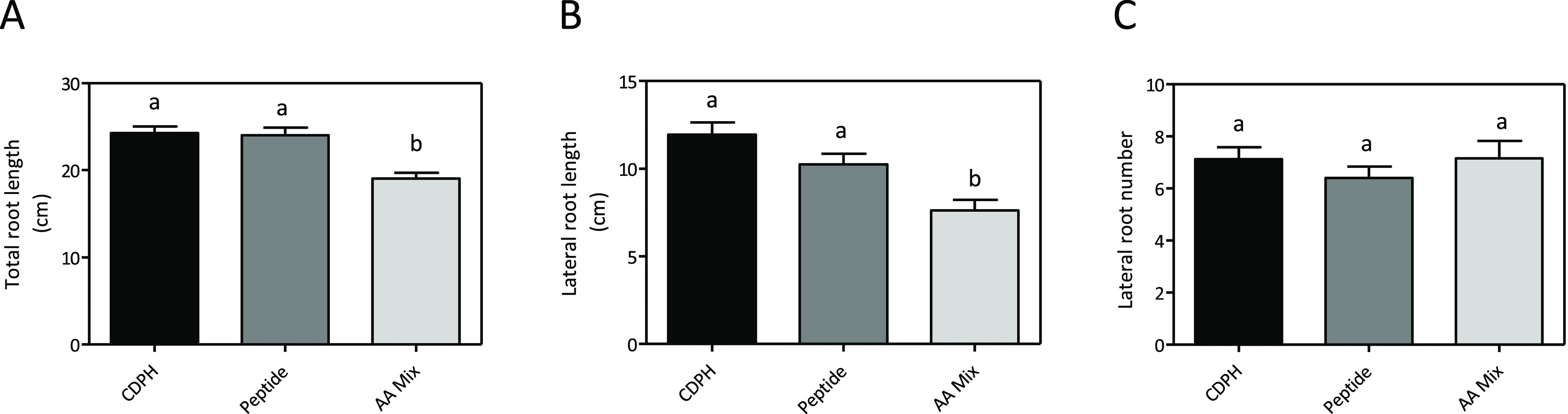
Effects of the CDPH compared to the synthetic peptide and an AA mixture on root growth of tomato seedlings *in vitro* agar media. Total root length (a), lateral root length (b), and lateral root number (c) of tomato seedlings treated with CDPH (total N 1.4 mg·L^–1^) and seedlings treated with synthetic peptide and free AA mixture containing an equal amount of total N. The seedlings were grown for 6 days in 8 g·L^–1^ agar plates. Root length was measured with WinRHIZO software. Mean values per plant are reported. Bars represent the standard error of the mean (*n* ≥ 19 replicates, one-way ANOVA with Tukey’s post hoc test, *p* < 0.05, significant differences are indicated by different letters). Here, we show the merged data from two independent replicates.

The biostimulant activity of the synthetic peptide was evaluated on the root phenotype of tomato seedlings grown *in vitro* in agar plates. In particular, as a first experiment, we compared its effect with those obtained by treating the seedlings with the CDPH or with an AA mixture mimicking the composition of the peptide (Figure 6). The experiment was carried out applying either the peptide, CDPH, or AAs at the same N rate used for the assessment of CDPH fraction effects (1.43 mg·L^–1^, Figure 3). The peptide stimulated root growth as much as the CDPH showed comparable values for lateral and total root length and lateral root number (Figure 6A–C). Plants treated with the AA mixture displayed a lower stimulatory effect on total and lateral root length if compared to peptide and CDPH treatment (Figure 6A,B), whereas no significant differences were observed in terms of number of lateral roots (Figure 6C).

**Figure 7:**
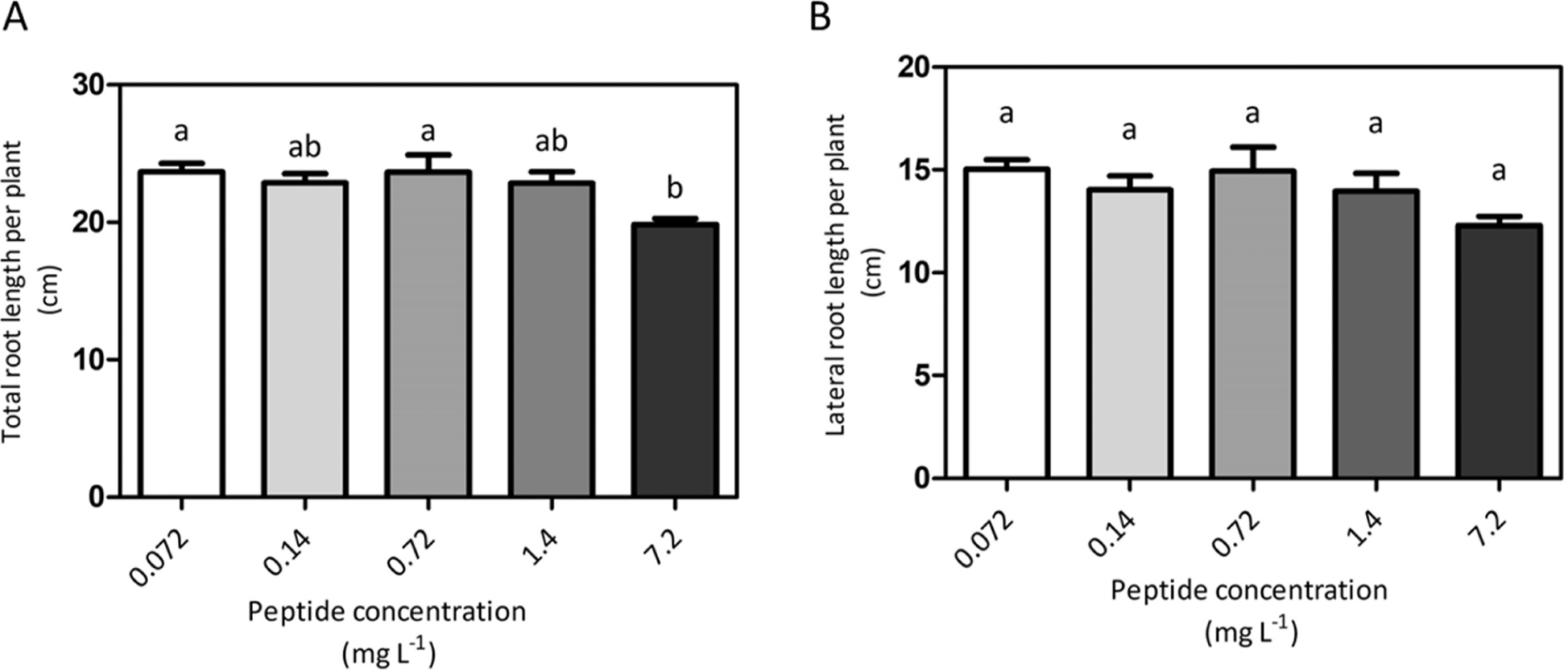
Effects of different concentrations of the synthetic peptide on root growth of tomato seedlings *in vitro* agar media. Total seminal and primary root (a) and lateral root length (b) after 6 days of culture of tomato seedlings treated with increasing concentration of the synthetic peptide containing total N of 0.072, 0.14, 0.72, 1.4, and 7.2 mg·L^–1^, respectively. Root length was measured with WinRHIZO software. Mean values per plant are reported. Bars represent the standard error of the mean (*n* ≥ 15 replicates, one-way ANOVA with Tukey’s post hoc test, *p* < 0.05, significant differences are indicated by different letters). Here, we show one experiment representative of three replicates.

The effect of different concentrations of the peptide (containing total N from 0.072 to 7.2 mg·L^–1^) on root growth was then assessed using the same experimental setup (Figure 7). We observed that the total and lateral root length did not increase in the range of total N concentrations from 0.072 to 1.4 mg·L^–1^ (Figure 7A,B). At a higher concentration (total N 7.2 mg·L^–1^), the effect of the peptide on total root growth was slightly reduced (Figure 7A). On the other hand, the number of lateral roots, counted after 6 days of treatment showed a tendency to increase as the peptide concentration increases from 0.072 to 7.2 N mg·L^–1^ (Supporting Information, Figure 5). This suggests a dose response effect of the peptide on lateral root formation, which is in agreement with our previous results obtained with the CDPH.^8^

## Discussion

The use of PHs in the agricultural practice to improve crop performance and resilience has dramatically increased in recent years as also evidenced by the rapid growth of their market. PHs are usually produced from agricultural or industrial wastes as raw materials, thus representing a good example of circular economy. PHs are also suitable for sustainable and organic agriculture as they are products active on plants at low concentrations, easily degraded in the environment and with beneficial effects on soil microbiota.^31,32^

The numerous scientific papers describing the stimulatory activity of PHs on plant growth, mineral nutrient uptake and assimilation as well as protection against abiotic stress factors have highlighted the efficacy of PHs on a wide range of crops and their multifunctional ability to affect many physiological processes.^6,33^ Despite the wealth of descriptive information on the beneficial effects of PHs, their mechanisms of action have been only partially addressed, partly in view of the intriguing fact that they exert their activity at concentrations on the order of mg·L^–1^, thus usually excluding a simple nutritional effect. One proposed mode of action involves PHs having hormonal activity similar to that exerted by certain endogenous signaling peptides. Peptides present in PHs could act as agonist or antagonist for endogenous peptide receptors by modulating downstream signaling pathways. In plants, many endogenous peptides possess hormonal activity and are potent local and systemic regulators of developmental processes such as, among others, root development and plasticity.^34,35^ In this regard, modulation of root growth and architecture is one of the main effects observed when PHs are supplied to plant by root drenching.^18^

One of the possible approaches to identify the bioactive compounds of PHs and their mechanisms of action is to apply fractionation methods to isolate different components and test their effects on plants. In the work of Lucini *et al.* (2020),^15^ a PH obtained from legume seed flour by enzymatic hydrolysis was separated by dialysis obtaining fractions containing molecules of different sizes. They tested the biostimulant activity of the fractions on tomato cuttings and found that the fractions containing free AAs and shorter oligopeptides (up to 1000 Da) were the fractions promoting root length the most. Analysis of the metabolomic profile of cuttings treated with the product containing shorter peptides and free AAs suggested that the response to PH1 mainly involved changes in phytohormone and secondary metabolism.

The results obtained in our work using a similar separation method (dialysis or filtration and dialysis) are in agreement with the results reported by Lucini *et al.*, 2020. Indeed, removal of molecules with a MW < 1000 Da significantly reduced the stimulatory effects of the CDPH on maize lateral root growth, whereas the >3000 Da fraction did not contribute to this effect. However, removal of the longest peptides slightly reduced the action of the CDPH on seminal and principal root growth in terms of both total length and surface area. These findings indicate that peptides of different lengths can exert specific biological effects on root development and we can hypothesize that by tailoring the peptide profile of a PH, it would be possible to achieve the desired change in root architecture. This would be of great importance to improve the crop response to water shortage and/or nutrient stress.^36,37^

We applied a more powerful fractionation method using size-exclusion chromatography to collect fractions that represent the most abundant peptide species of the CDPH. The FPLC profile of the CDPH showed that its prominent components consisted of peptides ranging from about 200 to 1400/1500 Da in size, thus confirming what was observed from the analysis of the biological effects of the fractions obtained by membrane filtration (Figure 1). The chromatographic profile of CDPH proved to be very stable, as changing the dilution of the product, or the batch of the product, and repeating the analysis after weeks/months of storage did not alter the shape of the profile (data not shown). These observations confirm that the CDPH maintains its integrity over a long period of time and that the industrial production process is characterized by a high reproducibility.

Chromatographic separation was also adopted to obtain samples suitable for MS analysis. In fact, preliminary MS analysis performed on unfractionated PH revealed that the complexity of the matrices, together with the high salt concentration of the elution buffer (PBS), severely limited the reliability of peptide identification (data not shown). By adopting sample fractionation and clean-up, 32 peptides were identified by MS, most of which in the FPLC fractions (26 identified peptides), indicating the importance of applying CDPH fractionation prior to MS analysis. To our knowledge, this is the first example of characterization of the peptide components of a CDPH. Matsumiya and Kubo (2011) identified a peptide with root hair promoting activity present in soybean meal treated with the degrading bacterium *Bacillus circulans* HA12.^17^ However, in that case they did not analyze the population of peptides present in the soybean meal, but isolated a single protein of the mixture, the Kunitz trypsin inhibitor, and analyzed by MS the degraded products obtained from the action of a single protease of *B. circulans* HA12.

The information about the AA sequence of the peptides in CDPH determined in our work provides much information about both the original raw material and the effects of the hydrolytic process. In particular, the analysis indicates that almost all identified peptides derive from a single type of protein, collagen. In addition, their length varies from 5 to 18 AA residues, in agreement with the results of the fractionation analyses. Finally, the results suggest that hydrolysis occurs preferentially at specific sites in the proteins.

Dissecting the CDPH peptide profile gives us the chance to identify and study thoroughly the effects and the mechanism(s) of action of individual peptides. Collagen-derived peptides showed high similarities in their sequence due to the repetitive nature of this protein, but surprisingly peptides derived from other proteins in CDPH also possess similar features in their sequence.

The existence of a common conserved motif GFPGLPGP suggests that the biological activity of different peptides is associated with a specific AA sequence/structure, supporting the hypothesis that these peptides may target endogenous signaling pathways. A 15 AA peptide, containing the GFLGLPG sequence identified by MS analysis, was synthetically produced to investigate these hypotheses. The first observation highlighted by the spectrometric CD analysis of the QGLLGApGFLGLpGS peptide was that it can form a PPII type helix resembling the secondary structure of the CDPH and transitively of the collagen profile. Even though the peptide is quite short, the capacity to form ordered PPII helices is well-documented in peptides as short as seven alanine residues.^38^ Curiously, the polyproline type II helix has a misleading name indeed because many PPII structures do not contain proline as just mentioned; however, it is well known that protein–protein binding motifs are often enriched in proline,^39^ which is abundant in the CDPH and also present in the peptide we chose to characterize. PPII type helices are often found in binding sites of SH3 domains, playing a key role in signal transduction and protein complex assembly.

The preliminary biophysical characterization of the synthetic peptide, with a secondary structure belonging to a peculiar and important type of helix, was then followed by an assay to test its capacity to retain the stimulatory effect on root growth typical of the CDPH. When the synthetic peptide was supplied to tomato seedlings, it produced the same effects on root growth as the CDPH mixture normalized to the same amount of total N. Furthermore, the bioactivity of the peptide could not be attributed to the effect of its AA composition because a free AA mixture employed as a control was found incapable to stimulate root growth as the CDPH. The peptide was active at a total N concentration of 1.43 mg·L^–1^ which well falls within the range of concentrations characterizing a signaling activity.

In conclusion, the present work describes an efficient chemical analytical method to study complex peptide matrices such as PHs, providing the first evidence for the chemical nature of the peptides present in CDPHs and demonstration of the biological activity of the individual components. We also identified the conserved motif characterizing these peptides and their stimulatory effect on root growth. This discovery opens up the possibility to investigate the mechanism of action of CDPHs in more detail and in the future to apply this information to obtain CPHD with tailored effects on plant growth and performance. It is also of particular relevance that a peptide possessing bioactivity in plants is for the first time identified in a PH derived from an animal matrix.

## Supporting Information Available

Concentration of total and organic N in the whole CDPH product and in the CDPH fractions, statistical data about peptide identification by LC–ESI–MS/MS analysis, effect of CDPH on basil, lettuce, and tomato root growth, lateral root growth of tomato seedlings grown *in vitro* in a nutrient solution supplemented with CDPH, *B. taurus* protein sequences with the indication of the 18 reference peptides, comparison between the CD spectra of the three CDPH fractions, and effects of different concentrations of the synthetic peptide on root growth of tomato seedlings (PDF)

## Abbreviations Used

AA: amino acid mixture
CD: circular dichroism
CDPH: collagen-derived protein hydrolysate
FPLC: fast protein liquid chromatography
LC–ESI–MS/MS: liquid chromatography–electrospray ionization–tandem mass spectrometry
MS: Murashige and Skoog
PBS: phosphate-buffered saline
PGPR: plant growth promoting bacteria
PH: protein hydrolysate
PPII: polyproline-II
