# Supplementary material for: Chemical Characterization of a Collagen-Derived Protein Hydrolysate and Biostimulant Activity Assessment of Its Peptidic Components
Source: J Agric Food Chem. 2022 Aug 30;70(36):11201–11. doi: 10.1021/acs.jafc.2c04379 (PMC9479078; doi:10.1021/acs.jafc.2c04379)
Supplement: Supplementary file 1 — jf2c04379_si_001.pdf [file jf2c04379_si_001.pdf]

Supporting information

Chemical characterization of a collagen-derived protein hydrolysate and biostimulant activity assessment of its peptidic components.

Stefano Ambrosini[1], Bhakti Prinsi[2], Anita Zamboni[1], Luca Espen[2], Serena Zanzoni[3], Chiara Santi [1], Zeno Varanini [1], Tiziana Pandolfini[1]*

1 Department of Biotechnology, University of Verona, 37134 Verona, Italy

2 Department of Agricultural and Environmental Sciences - Production, Landscape, Agroenergy, Università degli Studi di Milano, 20133, Milan, Italy

3 Centro Piattaforme Tecnologiche, University of Verona,37134 Verona, Italy.

*e-mail tiziana.pandolfini@univr.it

Table. S1 Concentration of total and organic N in the whole CDPH product and in the CDHP fractions.

	Total N (mg L^{-1})	Organic N (mg L^{-1})
**CDPH**	12.4	10.4
**Dialysis**	5.3	4.7
**Filtration and dialysis**	3.6	3.1

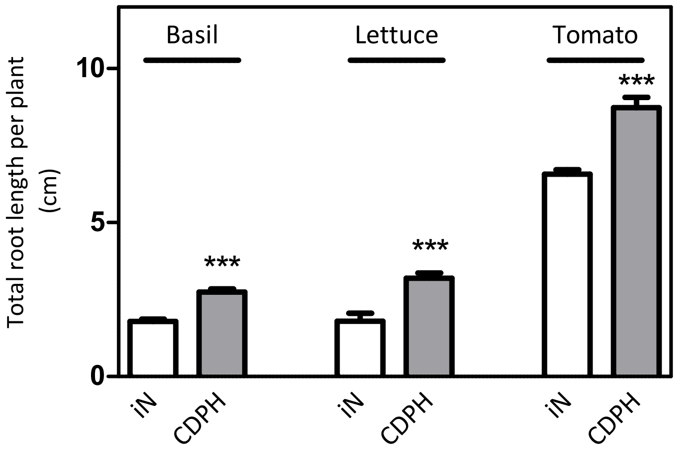

Figure S1 Effect of CDPH on basil, lettuce and tomato root growth. Total root length of basil, lettuce or tomato seedlings grown for 2 days on 8 g L^{-1} agar plates containing with CDHP (14.3 mg L^{-1}) or the equivalent amount of total inorganic N (iN). Root length was measured with WinRHIZO^{TM} software. Mean values per plant are reported. Error bars represent the standard error (SEM) [$n \geq 8$], Student's *t*-test was applied, ***p <0.001

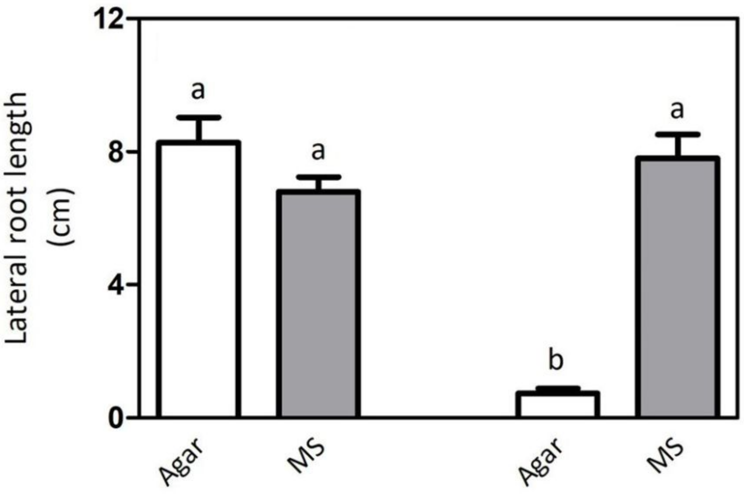

Figure. S2 Lateral root growth of tomato seedlings grown in vitro in a nutrient solution supplemented with CDPH. Total lateral root length of tomato seedlings grown in 8 g L^{-1} agar plates for 6 days. Seedlings were grown with $NH_4H_2PO_4$ added to reach a concentration of inorganic N of 1.43 mg L^{-1} (bars on the left) or 7.2 mg L^{-1} (bars on the right) in presence or absence of a 1:3 diluted MS solution dissolved in the agar gel. Root length was measured with WinRHIZO™ software. Mean values per plant are reported. Error bars represent the standard error (SEM) (n ≥ 12 replicates; one-way ANOVA with Tukey's post hoc test, $p < 0.05$, significant differences are indicated by different letters).

Table S2 Statistical data about peptide identification by LC-ESI-MS/MS analysis.

Peptide	z	Score	SPI (%)	Spectrum Intensity	MH^+ Matched (Da)	MH^+ Error (ppm)	Accession #	Protein Name
				**Unfractionated CDHP**				
**(R)GEVGLpGI(K)**	1	12.76	85.4	2.08E+06	741.414	0.1	A6QPB3	Collagen alpha-1(XVII) chain
**(G)GYEFGF(D)**	1	12.49	81.7	3.29E+05	719.303	3.7	P02465	Collagen alpha-2(I) chain
**(N)AVFEGp(A)**	1	11.74	80.7	4.36E+06	619.309	2.6	P81019	Seminal plasma protein BSP-30 kDa
**(G)FLPEPP(S)**	1	10.81	85.4	4.03E+05	699.371	0.7	Q8MJI9	Homeobox protein prophet of Pit-1
**(R)GIpGEFGLpG(P)**	1	10.81	89.7	3.61E+05	943.488	0.2	P02465	Collagen alpha-2(I) chain
**(T)GIPGMPG(S)**	1	10.00	89.3	2.02E+06	628.312	5.6	Q7SIB2	Collagen alpha-1(IV) chain
				**Fraction 1 (F1)**				
**(R)GVpGPpGAVGPA(G)**	2	19.84	94.9	6.27E+04	975.526	3.5	P02453	Collagen alpha-1(I) chain
**(R)GIpGEFGLpGPA(G)**	2	14.38	77.4	3.19E+04	1111.578	1.1	P02465	Collagen alpha-2(I) chain
**(R)GVpGpPGAVGPAGKDGEA(G)**	2	13.11	80.7	7.70E+05	1532.77	-1.1	P02453	Collagen alpha-1(I) chain
**(L)GApGFLGLpG(S)**	1	11.89	80.5	7.29E+05	885.483	0	P02465	Collagen alpha-2(I) chain
**(Q)AFLppPP(L)**	1	11.87	89.9	3.88E+05	738.418	0.5	Q9TTS3	Acetyl-CoA carboxylase 1
**(R)GLVGEpGPA(G)**	1	10.32	80.9	2.81E+06	796.42	-0.9	P02465	Collagen alpha-2(I) chain
				**Fraction 2 (F2)**				
**(G)GYEFGFD(G)**	1	15.28	76.6	1.38E+06	834.331	-0.3	P02465	Collagen alpha-2(I) chain
**(R)GFPGLpGP(S)**	1	15.13	89.9	5.37E+05	741.393	0.8	P02453	Collagen alpha-1(I) chain
**(P)GFVGEKG(P)**	2	14.51	89.9	4.43E+05	693.357	3.6	P02465	Collagen alpha-2(I) chain
**(G)GYEFGFDG(D)**	1	13.68	83.0	1.29E+06	891.352	-0.8	P02465	Collagen alpha-2(I) chain
**(P)LGpGEF(E)**	1	13.62	88.9	2.12E+06	619.309	1.7	Q0P5F7	Phosphatidylinositol 5-phosphate 4-kinase type-2 gamma
**(F)GFDGDFY(R)**	1	13.33	84.0	4.17E+05	820.315	0	P02465	Collagen alpha-2(I) chain
**(G)YEFGFD(G)**	1	13.30	88.9	4.85E+05	777.309	0.8	P02465	Collagen alpha-2(I) chain
**(G)FVGEKGP(S)**	2	13.11	81.2	1.34E+05	733.388	3.8	P02465	Collagen alpha-2(I) chain
**(Y)FGpGLGp(I)**	1	11.67	91.1	2.15E+06	644.34	1.2	P30205	Antigen WC1.1
**(A)GFAGPpG(A)**	1	11.65	82.7	5.82E+05	602.293	-3.6	P02453	Collagen alpha-1(I) chain
**(N)AVFEGp(A)**	1	11.53	76.7	2.57E+06	619.309	1.4	P81019	Seminal plasma protein BSP-30 kDa
**(K)GFpGIpG(Q)**	1	11.40	89.0	2.13E+06	644.34	1.1	Q7SIB2	Collagen alpha-1(IV) chain
**(P)GFLGLpG(S)**	1	11.19	82.0	2.43E+06	660.371	1.1	P02465	Collagen alpha-2(I) chain
**(A)pGFLGLpG(S)**	1	10.99	89.1	7.23E+05	757.424	0.1	P02465	Collagen alpha-2(I) chain
**(K)GFPGIp(G)**	1	10.94	84.0	1.11E+06	587.319	-0.1	Q7SIB2	Collagen alpha-1(IV) chain
**(N)FGGLLGp(L)**	1	10.43	82.2	4.79E+06	660.371	0.1	Q5QQ49	Xylosyltransferase 2
**(P)GFpGARGP(S)**	2	10.19	78.2	1.32E+05	758.394	2.9	P02453	Collagen alpha-1(I) chain
				**Fraction 3 (F3)**				
**(G)GYEFGF(D)**	1	15.70	91.8	1.12E+06	719.303	-0.4	P02465	Collagen alpha-2(I) chain
**(G)YEFGF(D)**	1	11.90	87.4	9.85E+05	662.282	-0.2	P02465	Collagen alpha-2(I) chain
**(G)GGYEFGF(D)**	1	10.92	75.5	3.29E+05	776.325	-1.3	P02465	Collagen alpha-2(I) chain

The table reports values referred to the best peptide hit identified among all the analyses. **Peptide**: amino acid sequence, p = hydroxyproline. **z**: charge state detected. **Score**: Spectrum Mill peptide score. **SPI (%)**: percentage score peak intensity. **MH$^+$ Matched**: mass of the ionized precursor (Da). **MH$^+$ Error**: difference between predicted and observed mass of the ionized precursor (ppm). **Accession #:** protein ID in UniProt database, for complete protein sequence see Supplementary Figure 3. Each sample was independently analyzed twice, and only peptides identified in both analyses were accepted. The search was done against the database of *Bos taurus* reviewed protein sequences downloaded from UniProt (https://www.uniprot.org/) (6003 entries, October 2018).

Figure S3 ***Bos taurus*** **protein sequences with the indication of the 18 reference peptides**

The reference peptides are highlighted in red and underlined

Collagen alpha-2(I) chain (P02465)

MLSFVDTRTLLLLAVTSCLATCQSLQEATARKGPSGDRGPRGERGPPGPPGRDGDDGIPGPPGPPGPPGPPGLGGNFAAQFDAKGGGPGPMGLMGP
RGPPGASGAPGPQGFQGPPGEPGEPGQTGPAGARGPPGPPGKAGEDGHPGKPGRPGERGVVGPQGARGFPGTPGLPGFKGIRGHNGLDGLKGQP
GAPGVKGEPGAPGENGTPGQTGARGLPGERGRVGAPGPAGARGSDGSVGPVGPAGPIGSAGPPGFPGAPGPKGELGPVGNPGPAGPAGPRGEVGL
PGLSGPVGPPGNPGANGLPGAKGAAGLPGVAGAPGLPGPRGIPGPVGAAGATGAR**GLVGEPGPA**GSKGESGNKGEPGAVGQPGPPGPSGEEGKRG
STGEIGPAGPPGPPGLRGNPGSRGLPGADGRAGVMGPAGSRGATGPAGVRGPNGDSGRPGEPGLMGPRGFPGSPGNIGPAGKEGPVGLPGIDGRP
GPIGPAGARGEPGNIGFPGPKGPSGDPGKAGEKGHAGLAGARGAPGPDGNNGAQGPPGLQGVQGGKGEQGPAGPPGFQGLPGPAGTAGEAGKPG
ER**GIPGEFGLPGPA**GARGERGPPGESGAAGPTGPIGSRGPSGPPGPDGNKGEPGVVGAPGTAGPSGPSGLPGERGAAGIPGGKGEKGETGLRGDIGSP
GRDGARGAPGAIGAPGPAGANGDRGEAGPAGPAGPAGPRGSPGERGEVGPAGPNGFAGPAGAAGQPGAKGERGTKGPKGENGPVGPTGPVGAA
GPSGPNGPPGPAGSRGDGGPPGATGFPGAAGRTGPPGPSGISGPPGPPGPAGKEGLRGPRGDQGPVGRSGETGASGPP**GFVGEKGP**SGEPGTAGPP
GTPGPQGLL**GAPGFLGLPG**SRGERGLPGVAGSVGEPGPLGIAGPPGARGPPGNVGNPGVNGAPGEAGRDGNPGNDGPPGRDGQPGHKGERGYPG
NAGPVGAAGAPGPQGPVGPVGKHGNRGEPGPAGAVGPAGAVGPRGPSGPQGIRGDKGEPGDKGPRGLPGLKGHNGLQGLPGLAGHHGDQGAPG
AVGPAGPRGPAGPSGPAGKDGRIGQPGAVGPAGIRGSQGSQGPAGPPGPPGPPGPPGPSG**GGYEFGFDGDFY**RADQPRSPTSLRPKDYEVDATLKSL
NNQIETLLTPEGSRKNPARTCRDLRLSHPEWSSGYYWIDPNQGCTMDAIKVYCDFSTGETCIRAQPEDIPVKNWYRNSKAKKHVWVGETINGGTQFEY
NVEGVTTKEMATQLAFMRLLANHASQNITYHCKNSIAYMDEETGNLKKAVILQGSNDVELVAEGNSRFTYTVLVDGCSKKTNEWQKTIIEYKTNKPSRL
PILDIAPLDIGGADQEIRLNIGPVCFK

Collagen alpha-1(I) chain (P02453)

MFSFVDLRLLLLLAATALLTHGQEEGQEEGQEEDIPPVTCVQNGLRYHDRDVWKPVPCQICVCDNGNVLCDDVICDELKDCPNAKVPTDECCPVCPEG
QESPTDQETTGVEGPKGDTGPRGPRGPAGPPGRDGIPGQPGLPGPPGPPGPPGPPGLGGNFAPQLSYGYDEKSTGISVPGPMGPSGPRGLPGPPGAP
GPQGFQGPPGEPGEPGASGPMGPRGPPGPPGKNGDDGEAGKPGRPGERGPPGPQGARGLPGTAGLPGMKGHRGFSGLDGAKGDAGPAGPKGEP
GSPGENGAPGQMGPRGLPGERGRPGAPGPAGARGNDGATGAAGPPGPTGPAGPPGFPGAVGAKGEGGPQGPRGSEGPQGVRGEPGPPGPAGAA
GPAGNPGADGQPGAKGANGAPGIAGAP**GFPGARGP**SGPQGPSGPPGPKGNSGEPGAPGSKGDTGAKGEPGPTGIQGPPGPAGEEGKRGARGEPG
PAGLPGPPGERGGPGSRGFPGADGVAGPKGPAGERGAPGPAGPKGSPGEAGRPGEAGLPGAKGLTGSPGSPGPDGKTGPPGPAGQDGRPGPPGPP
GARGQAGVMGFPGPKGAAGEPGKAGER**GVPGPPGAVGPAGKDGEA**GAQGPPGPAGPAGERGEQGPAGSPGFQGLPGPAGPPGEAGKPGEQGV
PGDLGAPGPSGARGERGFPGERGVQGPPGPAGPRGANGAPGNDGAKGDAGAPGAPGSQGAPGLQGMPGERGAAGLPGPKGDRGDAGPKGADG
APGKDGVRGLTGPIGPPGPAGAPGDKGEAGPSGPAGPTGARGAPGDRGEPGPPGPA**GFAGPPG**ADGQPGAKGEPGDAGAKGDAGPPGPAGPAGP
PGPIGNVGAPGPKGARGSAGPPGATGFPGAAGRVGPPGPSGNAGPPGPPGPAGKEGSKGPRGETGPAGRPGEVGPPGPPGPAGEKGAPGADGPAG
APGTPGPQGIAGQRGVVGLPGQRGER**GFPGLPGP**SGEPGKQGPSGASGERGPPGPMGPPGLAGPPGESGREGAPGAEGSPGRDGSPGAKGDRGET
GPAGPPGAPGAPGAPGPVGPAGKSGDRGETGPAGPAGPIGPVGARGPAGPQGPRGDKGETGEQGDRGIKGHRGFSGLQGPPGPPGSPGEQGPSGA
SGPAGPRGPPGSAGSPGKDGLNGLPGPIGPPGPRGRTGDAGPAGPPGPPGPPGPPGPPSGGYDLSFLPQPPQEKAHDGGRYYRADDANVVRDRDLE
VDTTLKSLSQQIENIRSPEGSRKNPARTCRDLKMCHSDWKSGEYWIDPNQGCNLDAIKVFCNMETGETCVYPTQPSVAQKNWYISKNPKEKRHVWYG
ESMTGGFQFEYGGQGSDPADVAIQLTFLRLMSTEASQNITYHCKNSVAYMDQQTGNLKKALLLQGSNEIEIRAEGNSRFTYSVTYDGCTSHTGAWGKT
VIEYKTTKTSRLPIIDVAPLDVGAPDQEFGFDVGPACFL

Collagen alfa 1 (IV) (Q7SIB2)

MGPRLGVWLLLLLAALLLHEESSRAAAKGGCAGSGCGKCDCHGVKGQKGERGLPGLQGVIGFPGMQGPEGPQGPPGQKGDTGEPGLPGTKGTRGPS
GVPGYPGNPGLPGIPGQDGPPGPPGIPGCNGTKGERGPVGPPGLPGFAGNPGPPGLPGMKGDPGEILGHIPGTLLKGERGYPGQPGAPGSPGLPGLQ
GPVGPPGFTGPPGPPGPPGPPGEKGQMGLSFQGPKGEKGDQGVSGPPGLPGQAQVITKGDTAMRGEKGQKGEPGFPGLPGFGEKGEPGKPGPRGK
PGKDGEKGEKGSPGFPGDSGYPGQPGQDGLKGEKGEAGPPGLPGTVIGTGPLGEKGEPGYPGGPGAKGETGPK**GFPGIPG**QPGPPGFPTPGLIGAPG
FPGDRGEKGEPGLPGVSLPGPSGRDGLPGPPGPPGPPGQPGHTNGIVECQPGPPGDQGPPGIPGQPGLTGEVGEKGQKGDSCLVCDTAELRGPPGPG
PPGEIGFPGQPGAKGDRGLPGRDGLEGLPGPQGAPGLMGQPGAKGEPGEIYFDIRLKGDKGDPGFPGQPGMPGRAGSPGRDGQPGLPGPRGSPGS
GLKGERGPPGGVGFPGSRGDIGPPGPPGFGPIGPIGDKGQIGFPGTPGAPGQPGPKGEAGKVVPLPGPPGAEGLPGSPGFQGPQGDRGFPGSPGRPG
LPGEKGAIGQPGIGFPGPPGPKGVDGLPGDAGPPGNPGRQGFNGLPGNPGPPGQKGEPGVGLPGLKGLPGIPGIPGTPGEKGNVGGPGIPGEHGAIG
PPGLQGLRGDPGPPGFQGPKGAPGVPGIGPPGAMGPPGGQGPPGSSGPPGVKGEKGFPGFPGLDMPGPKGDKGSQGLPGLTGQSGLPGLPGQQG
TPGQPGIPGPKGEMGVMGTPGQPGSPGPAGVPGLPGAKGDHGFPGSSGPRGDPGFKGDKGDVGLPGKPGSMDKVDMGSMKGEKGDQGEKGQT
GPTGDKGSRGDPGTPGVPGKDGQAGHPGQPGPKGDPGVSGIPGAPGLPGPKGSAGGMGLPGMPGPKGVAGIPGPQGIPGLPGDKGAKGEKGQAG

LPGIGIPGRPGDKGDQGLAGFPGSPGEKGEKGST**GIPGMPG**SPGPKGSPGSVGYPGSPGLPGEKGDKGLPGLDGIPGIKGEAGLPGKPGPTGPAGQKG
EPGSDGIPGSVGEKGESGLPGRGFPGFPGSKGDKGSKGDVGFPGLSGSPGIPGSKGEQGFMGPPGPQGQPGLPGTPGHAVEGPKGDRGPQGQPGLP
GRPGPMGPPGLPGLEGLKGERGNPGWPGTPGAPGPKGDPGFQGMPGIGGSPGITGAKGDVGPPGVPGFHGQKGAPGLQGVKGDQGDQGFPGTK
GLPGPPGPPGPFSIIKGEPGLPGPEGPAGLKGLQGPPGPKGQQGVTGSVGLPGPPGEPGFDGAPGQKGETGPFGPPGPRGFPGPPGPDGLPGSMGPP
GTPSVDHGFLVTRHSQTTDDPQCPPGTKILYHGYSLLYVQGNERAHGQDLGTAGSCLRKFSTMPFLFCNINNVCNFASRNDYSYWLSTPEPMPMSMA
PITGENIRPFISRCAVCEAPAMVMAVHSQTIQIPQCPTGWSSLWIGYSFVMHTSAGAEGSGQALASPGSCLEEFRSAPFIECHGRGTCNYYANAYSFWL
ATIERSEMFKKPTPSTLKAGELRTHVSRCQVCMRRT

Collagen alfa 1(XVII) (A6QPB3)

MDITQKNKRDGTEVTERIITETVTTRLTSLPPKGGTSNGYAKTGSLGGGSRLEKQSLTHGSSGYINSSGSLRGNASTSSYRRAHSPASTLPNSPGSTFERKT
HVTRHGTYEGSSSGNSSPEYPRKEFASSSTRGRSQTRESEIRVRLQSASPSTRWTELDDVKRLLKGSRSASVSPTRNSSNTLPIPKKGTVETKVVTASSQSVS
GTYDTTILDANLPSHVWSSTLPAGSSMGTYHNNITTQSSSLLNTNAYSAGSVFGVPNNMASCSATLQPGISTSSSVFGMQNNLAPSSSTLSHGMAATST
AYGVKKNMPQSPTAVSTGVSTSAASTTNVQNDDLLHKDCKFLILEKDNTPAKKEMELLIMTKDSGKVFTASPASVAATSFSEDTLKKEKQAAYTDTYLVS
EANGDVKTVTAKGNGASADIHGYDHRRGGGGGGGSGGALGSGAAGGGGKGSWGAAPTWCPCGSWCSWWKWLLGLLLTWLLLLGLLFGLIALAEE
VRKLKARVEELEKMRGRLSYNEKMERSSQDSVQGVAPRLGEGLGKSELDDYNLEDVWQFMKVRLMTEQENGNLRGSPGPKGDMGVQGPKGDRGF
PGTPGIPGPLGHQGPEGPKGQKGNVGEPGMEGPMGQRGREGPMGPRGEPGPPGFGEKGDRGDAGKPGIPGPPGVPGSVGPKGSIGPQGLR**GEVG**
LPGIKGDKGPMGPPGPKGDQGEKGPRGLTGEPGLKGLPGAVGEPGAKGAMGPAGPDGHQGPRGEQGLPGMPGTRGLPGPSGDPGKPGLTGPQGP
QGIPGTPGRPGVKGEPGAPGKIMTSEGSSTITVPGPPGPPGAMGPPGPPGAPGPVGPAGLPGQQGPRGEPGLAGESFMGSSSSFSEVLSTQGIDLRGP
PGPPGPPGPPGEGLPGPPGPPGSLLTSSETFFSGPPGPPGPPGPKGDQGPPGPRGHQGERGFPGLSGSGSSSLGLNLQGPPGPPGPQGPKGDKGDPG
VPGAPGIPGGPSRGGSSSSTTFMQGPPGPPGPPGPPGSLSSSGLEIQQYISDYMQSDSIRPYLSGVQGPPGPPGPPGPVTTITGETFNYSELASLVVSYLQ
TSGYNIGTSSTSISSEDILAALRRDDVRQYLQQYLMPQGAGGDWFLQSLDYAELSNRILSYMSSTGVSIGLPGPPGPPGLPGTSYEELLSLLQGSEFRGIVG
PPGPPGPPGLPGSSWSSISTEDLSSYLQTAGLSSIPGPPGPPGPPGPRGPPGISGALATYAAENSDSFRSELISYLTSPDVRSFIVGPPGPPGPQGPPGDTRL
VSTDSSYSRSGSSSSFSRDTSYSSSMGIGGASGGSLGEAGAFGMDMGRGYGAAAESGMYGGNGRFGTSFAGGLDYNELAVRVSESLQRQGLLQGMA
YTVQGPPGRPGPQGPPGISKIFSAYSNVTEDLMDFFRTYGAIPGPPGQKGEMGIPGPKGERGPAGPPGPRGHKGEKGDKGDQFYIGRRRRSIAVKP

Seminal plasma protein (P81019)

MAPLVGLFLIWAGASVFQQLHPVNGGDIPDPGSKPTPPGMADELPTETYDLPPEIYTTTFLPRTIYPQEEMPYDDKPFPSLLSKANDLN**AVFEGP**ACAFP
FTYKGKKYYMCTRKNSVLLWCSLDTEYQGNWKFCTERDEPECVFPFIYRKKSYESCTRVHSFFWRRWCSLTSNYDRDKAWKYC

Homeobox protein (Q8MJI9)

MDTEGSSEQGKQTKEQVCSSLWPEGYPAAETVTSSVDMNTQPYRNLSGVRVGRPKLSLQGVQRGRPHSRRRHRTTFSPAQLEQLESAFGRNQYPDIW
ARESLAQDTGLSEARIQVWFQNRRAKQRKQERSLLQPLAHLSPATFSG**FLPEPP**SCPYSYPTPPPPMTCFPHPYNRALPSQPSTGSSFARPYQSEDWYPN
LHPTPAGHLACPPPPPMLPLSLEPPKSWN

Acetyl-CoA carboxylase (Q9TTS3)

MDEPSSLAKPLELNQHSRFIIGSVSEDNSEDEISNLVKLDLLEEKEGSLSPASVSSDTLSDLGISSLQDGLALHMRSSMSGLHLVKQGRDRKKIDSQRDFTV
ASPAEFVTRFGGNKVIEKVLIANNGIAAVKCMRSIRRWSYEMFRNERAIRFVVMVTPEDLKANAEYIKMADHYVPVPGGPNNNNYANVELILDIAKRIP
VQAVWAGWGHASENPKLPELLLKNGIAFMGPPSQAMWALGDKIASSIVAQTAGIPTLPWSGSGLCVDWHENDFSKRILNVPQELYEKGYVKDVDDG
LKAAEEVGYPVMIKASEGGGGKGIRKVNNADDFPNLFRQVQAEVPGSPIFVMRLAKQSRHLEVQILADQYGNAISLFGRDCSVQRRHQKIIEEAPAAIAT
PAVFEHMEQCAVKLARMVGYVSAGTVEYLYSQDGSFYFLELNPRLQVEHPCTEMVADVNLPAAQLQIAMGIPLYRIKDIRMMYGVSPWGDAPIDFEN
SAHVPCPRGHVIAARITSENPDEGFKPSSGTVQELNFRSNKNVWGYFSVAAAGGLHEFADSQFGHCFSWGENREEAISNMVVALKELSIRGDFRTTVEY
LIKLLETESFQLNRIGTGWLDRLIAEKVQAERPDTMLGVVCGALHVADVSLRNSISNFLHSLERGQVLTAHTLLNTVDVELIYEGVKYVLKVTRQSPNSYVV
IMNGSCVEVDVHRLSDGGLLLSYDVSSYTTYMKEEVDRYRITIGNKTCVFEKENDPSVLRSPSAGKLIQYIVEDGGHVFAGQCYAEIEVMKMVMTLTAAE
SGCIHYVKRPGAALDPGCVIAKMQLDNPSKVQQAELHTGSLPRIQSTALRGEKLHRVFHYVLDNLVNVMNGYCLPDPFFSSRVKDWVERLMKTLRDPS
LPLLELQDIMTSVSGRIPPNVEKSIKKEMAQYASNITSVLCQFPSQQIANILDSHAATLNRKSEREVFFMNTQSIVQLVQRYRSGIRGHMKAVVMDLLRQ
YLRVETQFQNGHYDKCVFALREENKSDMNTVLNYIFSHAQVTRKNLLVTMLIDQLCGRGPTLTDELLNILTELTQLSKTTNAKVALRARQVLIASHLPSYEL
RLNQVESIFLSAIDMYGHQFCIENLQKLILSETSIFDVLPNFFYHSNQVVRMAALEVYVRRAYIAYELNSVQHRQLKDNTCVVEFQFMLPTSHPNRGNIPT
LNRMSFSSNLNHYGMTHVASVSDVLLDNAFTPPCQRMGGMVSFRTFEDFVRIFDEVMGCFCDSPPQSPTFPEAGHTSLYDEDKVPRDEPIHILNVAIKT
DCDIEDDSLAAMFREFTQQNKATLVEHGIRRLTFLVAQKDFRKQVNYEVDQRFHREFPKFFTFRARDKFEEDRIYRHLEPALAFQLELNRMRNFDLTAIP
CANHKMHLYLGAAKVEVGTEVTDYRFFVRAIIRHSDLVTKEASFEYLQNEGERLLLEAMDELEVAFNNTNVRTDCNHIFLNFVPTVIMDPSKIEESVRSM
VMRYGSRLWKLRVLQAELKINIRLTPTGKAIPIRLFLTNESGYYLDISLYKEVTDSRTAQIMFQAYGDKQGPLHGMLINTPYVTKDQLQSKRFQAQSLGTT
YIYDIPEMFRQSLIKLWESMSSQ**AFLPPPP**LPSDILTYTELVLDDQGQLVHMNRLPGGNEIGMVAWKMTLKSPEYPDGRDIIVIGNDITYRIGSFGPQEDL
LFLRASELARAEGIPRIYVAANSGARIGLAEEIRHMFHVAWVDPEDPYKGYKYLYLTPQDYKRVSALNSVHCEHVEDEGESRYKITDIIGKEEGLGAENLRG
SGMIAGESSLAYDEIITISLVTCRAIGIGAYLVRLGQRTIQVENSHLILTGAGALNKVLGREVYTSNNQLGGIQIMHNNGVTHSTVCDDFEGVFTVLHWLSY
MPKSVYSSVPLLNSKDPIDRVIEFVPTKAPYDPRWMLAGRPHPTQKGQWLSGFFDYGSFSEIMQPWAQTVVVGRARLGGIPVGVVAVETRTVELSIPA
DPANLDSEAKIIQQAGQVWFPDSAFKTYQAIKDFNREGLPLMVFANWRGFSGGMKDMYDQVLKFGAYIVDGLRECSQPVMVYIPPQAELRGGSWVV
IDPTINPRHMEMYADRESRGSVLEPEGTVEIKFRRKDLVKTMRRVDPVYIHLAERLGTPELSVAERKELESKLKEREEFLLPIYHQVAVQFADLHDTPGRM

QEKGVINDILDWKTSRTFFYWRLRRLLLEDLVKKKIHNANPELTDGQIQAMLRRWFVEVEGTVKAYVWDNNKDLVEWLEKQLTEEDGVRSVIEENIKYI
SRDYVLKQIRSLVQANPEVAMDSIVHMTQHISPTQRAEVVRILSTMDSPST

Phosphatidylinositol 5-phosphate 4-kinase type-2 gamma (Q0P5F7)

MASSSVPPTAVPAATAAPGAGFGFASKTKKKHFVQQKVKVFRAADPLVGVFLWGVAHSINELSQVPPPVMLLPDDFKASSKIKVNNHLFHRENLPSHF
KFKEYCPQVFRNLRDRFGIDDQDYLVSLTRSPPTESEGSDGRFLISYDRTLVIKEVSSEDIADMHSNLSNYHQYIVKCHGNTLLPQFLGMYRVSVDSEDSY
MLVMRNMFSHRLPVHRKYDLKGSLVSREASDKEKVKELPTLKDMDFLNKNQKVYIGEEEKKVFLEKLKRDVEFLVQLKIMDYSLLLGIHDIIRGSEPEEDG
PVREEESEGDGDCGLTGPPAPVGSYGTSPEGIGGYIHSHRP**LGPGEF**ESFIDVYAIRSAEGAPQKEVYFMGLIDILTQYDAKKKAAHAAKTVKHGAGAEIS
TVHPEQYAKRFLDFITNIFA

Xylosyltransferase (Q5QQ49)

MVASARVQKLVRRYKLAIATALAILLLQGLVVWSFSVLEDDEPGEKGRQKKSRPLDPSEGSKDTDSSAGRRGSAGRRHGRWRGRAESPGVPVAKVVRA
VTSRHRTGRRIPPTPPPEAPGRQNLSGAAAEEALVGAAGFPHGDTGSVEGAPQPTDNSFTPKCEIVGKDALSALARASSKQCQQEIANVVCLHQAGSL
MPKAVPRHCQRAGKMSPGIPWDEVRAQQPADGPPVRIAYMLVVHGRAIRQLKRLLKAVYHKQHFFYVHVDERSNYLHREVVELARQYDNVRVTPW
RMVTIWGGASLLRMYLRSMQDLLEVPGWAWDFFINLSATDYPTRTNEELVAFLSKNRDKNFLKSHGRDNSRFIKKQGLDRLFHECDSHMWRLGERQI
PAGIVVDGGSDWFVLTRSFVEYVVYTDDPLVAQLRQFYTYTLLPAESFFHTVLEISPACESLVDNNMRVTTWNRKMGSKSQYKHIVDWCGCSPNDFKP
QDFLRLQQTARPTFFARKFEAVVNQEIIGQLDYYLYGNYPAGTPGLRSYWENVYDEPDGIHSLSDVTLTLYHSFSRLGLRRAEASLRAPGESSCRFEPRGLP
SSVHLYFYDDHFQGYLVTQAVQSSAQGPAETLEMWLMPQGSLKLLGHSDQASRLQSLEVGQVGTEWDPKERLFRN**FGGLLGP**LDEPVAMQRWARG
PNLTVTVVWIDPTYVVATSYDIVVDAETEVTQYKPPLSRPLRPGAWTVRLLQFWEPLGETRFLVLPLTFNRKLPLRKGKFSQMIAGPPHNEYMEQSFQGL
SGILNLPQLEPAEEAARLHAELTGPALEAWTDGEQSSFWSVAGVCAVGPSACPSLELCRLTSWSSLSPDPKSELGPVKADGRLR

Antigen WC1.1 (P30205)

MALGRHLSLRGLCVLLLGTMVGGQALELRLKDGVHRCEGRVEVKHQGEWGTVDGYRWTLKDASVVCRQLGCGAAIGFPGGAY**FGPGLGP**IWLLYTS
CEGTESTVSDCEHSNIKDYRNDGYNHGRDAGVVCSGFVRLAGGDGPCSGRVEVHSGEAWIPVSDGNFTLATAQIICAELGCGKAVSVLGHELFRESSAQ
VWAEEFRCEGEEPELWVCPRVPCPGGTCHHSGSAQVVCSAYSEVRLMTNGSSQCEGQVEMNISGQWRALCASHWSLANANVICRQLGCGVAISTPG
GPHLVEEGDQILTARFHCSGAESFLWSCPVTALGGPDCSHGNTASVICSGNQIQVLPQCNDSVSQPTGSAASEDSAPYCSDSRQLRLVDGGGPCAGRV
EILDQGSWGTICDDGWDLDDARVVCRQLGCGEALNATGSAHFGAGSGPIWLDNLNCTGKESHVWRCPSRGWGQHNCRHKQDAGVICSEFLALRM
VSEDQQCAGWLEVFYNGTWGSVCRNPMEDITVSTICRQLGCGDSGTLNSSVALREGFRPQWVDRIQCRKTDTSLWQCPSDPWNYNSCSPKEEAYIW
CADSRQIRLVDGGGRCSGRVEILDQGSWGTICDDRWDLDDARVVCKQLGCGEALDATVSSFFGTGSGPIWLDEVNCRGEESQVWRCPSWGWRQHN
CNHQEDAGVICSGFVRLAGGDGPCSGRVEVHSGEAWTPVSDGNFTLPTAQVICAELGCGKAVSVLGHMPFRESDGQVWAEEFRCDGGEPELWSCPR
VPCPGGTCLHSGAAQVVCSVYTEVQLMKNGTSQCEGQVEMKISGRWRALCASHWSLANANVVCRQLGCGVAISTPRGPHLVEGGDQISTAQFHCSG
AESFLWSCPVTALGGPDCSHGNTASVICSGNHTQVLPQCNDFLSQPAGSAASEESSPYCSDSRQLRLVDGGGPCGGRVEILDQGSWGTICDDDWDLD
DARVVCRQLGCGEALNATGSAHFGAGSGPIWLDDLNCTGKESHVWRCPSRGWGRHDCRHKEDAGVICSEFLALRMVSEDQQCAGWLEVFYNGTW
GSVCRSPMEDITVSVICRQLGCGDSGSLNTSVGLREGSRPRWVDLIQCRKMDTSLWQCPSGPWKYSSCSPKEEAYISCEGRRPKSCPTAAACTDREKLRL
RGGDSECSGRVEVWHNGSWGTVCDDSWSLAEAEVVCQQLGCGQALEAVRSAAFGPGNGSIWLDEVQCGGRESSLWDCVAEPWGQSDCKHEEDA
GVRCSGVRTTLPTTTAGTRTTSNSLPGIFSLPGVLCLILGSLLFLVLVILVTQLLRWRAERRALSSYEDALAEAVYEELDYLLTQKEGLGSPDQMTDVPDENY
DDAEEVPVPGTPSPSQGNEEEVPPEKEDGVRSSQTGSFLNFSREAANPGEGEESFWLLQGKKGDAGYDDVELSALGTSPVTFS

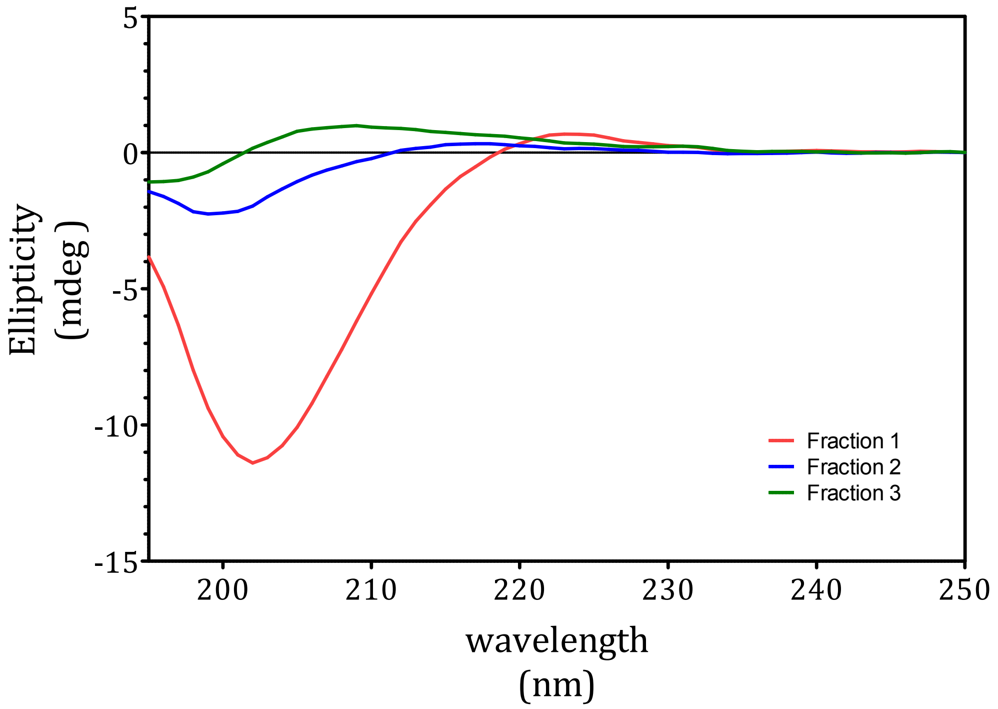

Figure S4 Comparison between the circular dichroism spectra of the three CDPH fractions: fraction 1 (red), fraction 2 (blue), fraction 3 (green) diluted in ½ PBS. Ellipticity is expressed as mdeg.

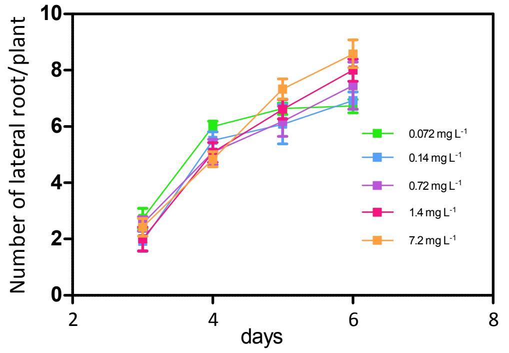

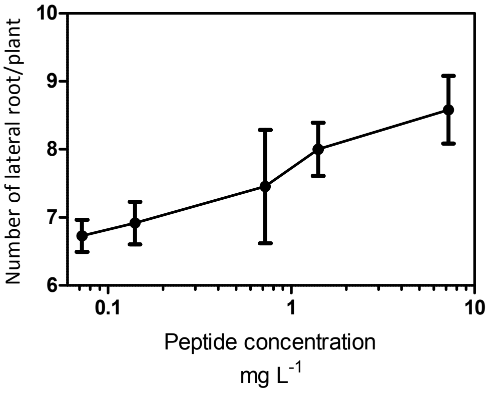

Figure S5 Effects of different concentrations of the synthetic peptide on root growth of tomato seedlings. Lateral root number progression over time (a) and at 6 days of culture (b) of tomato seedlings treated with 0.072, 0.14, 0.72, 1.4 and 7.2 mg· L^{-1} of the synthetic peptide. The seedlings were grown for 6 days in 0.8 g· L^{-1} agar plates. Root length was measured with WinRHIZO™ software. Mean values per plant are reported. Bars represent the standard error (SEM) (n ≥ 15 replicates).
